# Editorial: Artificial intelligence to enhance biomechanical modelling

**DOI:** 10.3389/fspor.2023.1188035

**Published:** 2023-04-28

**Authors:** S. David, B. Bačić, C. Richter, M. Mundt

**Affiliations:** ^1^ Department of Human Movement Sciences, Vrije Universiteit Amsterdam, Amsterdam, Netherlands; ^2^ School of Engineering, Computer and Mathematical Sciences, Auckland University of Technology, Auckland, New Zealand; ^3^ Sports Medicine Department, Sports Surgery Clinic, Dublin, Ireland; ^4^ Tech & Policy Lab, University of Western Australia, Perth, WA, Australia

**Keywords:** neural networks, wearable sensors, human movement, machine learning, video analysis

**Editorial on the Research Topic** Artificial intelligence to enhance biomechanical modelling

Artificial Intelligence (AI) has become substantial in information and data processing and has found its way into human movement science where it strives to revolutionise data collection and evaluation. Complementing laboratory-based data collection with in-field experiments is already possible. However, due to accuracy differences of low-cost data acquisition technology vs. standard laboratory equipment and the need for multidisciplinary collaboration, the uptake of AI in biomechanics is slower than expected. Therefore, the research topic — *Artificial Intelligence to Enhance Biomechanical Modelling* is aimed at inviting leading authors to help accelerate advancements in this field. The scope of the research topic targeted articles that depict the full range from state-of-the-art reporting to proof-of-concept studies as well as studies focussing on technological advancements that are promising in supporting the route to real-life, real-time use of AI in human movement science.

A selection of five articles has contributed to this research topic in terms of real-time modelling, the use of wearable and other motion capture technologies, AI modelling and data analysis, as well as the identification of motion patterns associated with specific activities.

The first article in this collection by Hammes et al*.* reviewed the current state-of-the-art of AI applications in elite sports. They found the usage of AI in signal processing, modelling, and feedback systems to be most promising. AI has been used to process signals of wearable sensors, e.g., collected by wrist-worn watches or smartphones, and computer vision techniques have been applied to analyse videos. Emerging deep learning and unsupervised learning methods have been used to model time-series data or detect motion patterns. To provide feedback, AI applications enable the development of smart clothing, which is on the rise, albeit still scarce. To further the use of AI in elite sports, the authors identified six challenges: data collection and sharing to achieve sufficient dataset sizes, user acceptance, explainability of AI results, robustness of predictive models, and user feedback.

In the second article, Noteboom et al*.* demonstrated and validated how video footage can be used to “see” into dimensions a practitioner cannot see. In their study, they used a convolutional neural network to estimate muscle forces of the upper limbs. The validation against the gold-standard procedure revealed strong to excellent correlations (*r* = 0.72–0.97) for the maximum force estimations of the main muscle groups. The use of AI and video data in this study give us a glimpse into a possible future where physicians and coaches evaluate movements and spot anomalies in a domain which will always be hidden from us. Such information can help to personalise the development of strength training or (p)rehabilitation, improving the current standard of care. Furthermore, utilising such a framework can not only streamline data capture but also introduce objective measures into the day-to-day practice of physicians and coaches.

The study by Mascia et al*.* presented a ready-to-use and wearable method to estimate jump height using a smartphone and reported that using a multilayer perceptron neural network improved the prediction accuracy 4.5-fold when compared to the jump height estimation using the time-of-flight only. This creates the opportunity to assess athletes in their real movement environment. Further, the low-cost application allows data collection to a broad field of users, therefore democratising research attempts. Moreover, this enables the creation of large datasets that can be shared and re-purposed.

Svensson et al*.* demonstrated the benefits of a non-invasive, easy-to-set-up data collection method to analyse muscle activation during barbell squats. The analysis presented can help to reveal motion patterns associated with squat technique suggesting follow-up unobtrusive wearable EMG data collection in real-world environments and near-future research on lower body movement analysis that could be extended to include various skill levels and common errors. The article is also aligned to a related secondary context of the journal's research topic, potentially pushing the boundaries of biomechanics regarding the near-future use of AI to process data from new sensor (textile EMG) technology to help athletes improve their footwork, stability and overall performance. Such articles could also contribute to dataset dissemination allowing computer scientists to contribute to scientific discoveries in human motion modelling and analysis.

Finally, considering the recent end-to-end processing trends in AI, which do not require multidisciplinary expertise for sophisticated task-oriented feature extraction engineering, Bach et al*.* used a reservoir computing approach to predict the vertical ground reaction force to detect gait events from acceleration data of wearable sensors attached to the participants' shanks. The produced Echo State Network (ESN) with 1,000 interconnected neurons achieved high prediction accuracy (>95%) on captured 3D sensor data. Practical implications of the study suggest broad applications in rehabilitation and various sport domains that could benefit from similar setups outside the lab using low-cost sensor technology. As an added contribution to the research community, the authors have provided links to the experimental dataset and the source code (https://github.com/marlow17/PredictingGroundReactionForces).

In conclusion, published proofs-of-concept and data/code-sharing contributions for the scientific community can accelerate the pace of global scientific and technological advancements for humanity in adding value to active lifestyles motor skill acquisition, sports performance, rehabilitation, and people living on their own. With a growing body of literature on human motion modelling and analysis, we can see promising applications of AI and recurrent neural networks such as ESN in advancing data stream pre-processing for real-time multi-dimensional time-series predictions and classification tasks such as event detection, indexing, phasing analysis and assessments of characteristic movement patterns. The use of end-to-end AI combined with traditional machine learning approaches also suggests future research opportunities for movement science in terms of real-time pre-processing and processing of data from multiple sources. All the inventions will bring objective measures into movement science and hence help improve training interventions.

The objective of this research topic has been achieved through the interdisciplinary application of AI, biomechanics and data acquisition technology. We anticipate that highly accurate laboratory technology will continue its role to obtain ground-truth data. Additionally, the opportunities to use low-cost data acquisition technology for in-field data collection will broaden the application of biomechanical modelling in human movement science for the benefit of society.

